# Local Activity and Causal Connectivity in Children with Benign Epilepsy with Centrotemporal Spikes

**DOI:** 10.1371/journal.pone.0134361

**Published:** 2015-07-30

**Authors:** Yun Wu, Gong-Jun Ji, Yu-Feng Zang, Wei Liao, Zhen Jin, Ya-Li Liu, Ke Li, Ya-Wei Zeng, Fang Fang

**Affiliations:** 1 Department of Neurology, Beijing Children’s Hospital Affiliated to Capital Medical University, Beijing, China; 2 Laboratory of Cognitive Neuropsychology, Department of Medical Psychology, Anhui Medical University, Hefei, China; 3 Center for Cognition and Brain Disorders and the Affiliated Hospital, Hangzhou Normal University, Hangzhou, China; 4 Zhejiang Key Laboratory for Research in Assessment of Cognitive Impairments, Hangzhou, China; 5 fMRI Center, The 306 Hospital of People’s Liberation Army, Beijing, China; University of Electronic Science and Technology of China, CHINA

## Abstract

The aim of the current study was to localize the epileptic focus and characterize its causal relation with other brain regions, to understand the cognitive deficits in children with benign childhood epilepsy with centrotemporal spikes (BECTS). Resting-state functional magnetic resonance imaging (fMRI) was performed in 37 children with BECTS and 25 children matched for age, sex and educational achievement. We identified the potential epileptogenic zone (EZ) by comparing the amplitude of low frequency fluctuation (ALFF) of spontaneous blood oxygenation level dependent fMRI signals between the groups. Granger causality analysis was applied to explore the causal effect between EZ and the whole brain. Compared with controls, children with BECTS had significantly increased ALFF in the right postcentral gyrus and bilateral calcarine, and decreased ALFF in the left anterior cingulate cortex, bilateral putaman/caudate, and left cerebellum. ALFF values in the putaman/caudate were positively correlated with verbal IQ scores in patients. The ALFF values in cerebellum and performance IQ scores were negatively correlated in patients. These results suggest that ALFF disturbances in the putaman/caudate and cerebellum play an important role in BECTS cognitive dysfunction. Compared with controls, the patients showed increased driving effect from the EZ to the right medial frontal cortex and posterior cingulate cortex and decreased causal effects from the EZ to left inferior frontal gyrus. The causal effect of the left inferior frontal gyrus negatively correlated with disease duration, which suggests a relation between the epileptiform activity and language impairment. All together, these findings provide additional insight into the neurophysiological mechanisms of epilepitogenisis and cognitive dysfunction associated with BECTS.

## Introduction

Benign childhood epilepsy with centrotemporal spikes (BECTS) (also known as rolandic epilepsy) is the most common form of idiopathic focal epilepsy syndrome in childhood [1]. BECTS is not correlated with any brain lesion and may be genetically determined. Its onset is between the ages of 1 and 14 years, with a peak at 7–10 years, usually followed by recovery during adolescence [2]. Given these characteristics, BECTS is classically considered a benign condition. However, there is accumulating evidence that BECTS can present with a variety of cognitive comorbidities including: language dysfunction [3–6], attention deficit [7–9], and difficulty with spatial perception [10] and memory and phonological awareness [11]. These findings suggest that seizures also affect cognitive function during interictal periods in children with BECTS, but the underlying mechanism of this cognitive impairment remains to be elucidated.

Resting-state functional magnetic resonance imaging (fMRI) has been used extensively to study functional brain activity in various types of epilepsy free of specifically designed behavioral tasks [12–17]. Amplitude of low-frequency fluctuation (ALFF) is a useful tool for depicting local brain activity. ALFF measures the magnitude of spontaneous blood oxygenation level dependent (BOLD) activity of each voxel; reflects brain activity level during a given period of time; and may be similar to positron emission tomography measurement [18,19]. In temporal lobe epilepsy, it has been demonstrated that ALFF is useful for localizing epileptic focus in the mesial temporal lobe, and other cortical and subcortical structures associated with cognitive impairment [20,21]. Additionally, increased ALFF is positively correlated with the number of epileptic discharges [20], and reflects the BOLD activation induced by epileptic activity [2]. We hypothesized that ALFF can identify the increased activity of the epileptic focus of BECTS, which is mainly located in the inferior part of the rolandic area.

ALFF can provide information regarding regional spontaneous activity, and functional connectivity measures the synchronicity of neuronal activity signals among regions of the brain. They may complement each other and provide more information about the underlying processes resulting in changes in resting-state brain function. It has recently been demonstrated that functional connectivity is reduced between the sensorimotor and language networks in children with BECTS [22,23]. Concordant abnormalities in structural connectivity have also been found in this type of childhood epilepsy [24]. Functional connectivity estimated the functional integrate between brain regions. But it ignored an important point to understand epilepsy—the direction of information flow. The abnormal driving effect from epileptogenic zone (EZ) to other functional areas could be a major reason of multiple clinic syndromes in epilepsy patients. Granger causality analysis (GCA) is a prominent technique for inferring the direction of information flow in brain networks [25–27]. It enabled us to understand better how seizure activity initiates, propagates and terminates [28].

The aim of the current study was to reveal the underlying mechanism of epileptogenesis and the cognitive deficit associated with BECTS. We adopted ALFF to identify the possible EZ of BECTS, and utilized GCA to test directly the direction and magnitude of influence between the EZ and other brain regions. All the observed abnormality would be correlated with cognitive estimations in BECTS patients.

## Materials and Methods

### Participants

This study involved 37 children with BECTS aged 7.1–13.5 years and 25 healthy volunteers aged 7.6–12.4 years, who were all attending regular schools. The mean and standard deviation of the ages in these two groups of children were, 9.8 ± 1.5 years and 10.0 ± 1.5 years, respectively. None of the healthy controls had a history of dyslexia, learning disorders or psychiatric disorders. There were no significant differences between patients and healthy controls for age (*P* = 0.522) or sex distribution (BECTS, 46% male; healthy controls, 60% male; *P* = 0.570). The inclusion criteria for patients were as follows: (1) diagnosed with BECTS according to the current diagnostic criteria [29]; (2) no other neurological disease; (3) no abnormality in routine structural MRI examinations; (4) aged 7–14 years; and (5) full-scale IQ (FSIQ) >70. Thirty six patients were right-handed and one was left-handed; 23 healthy controls were right-handed and two left-handed. Thirty-three patients had not received any antiepileptic drugs, three had received one antiepileptic drug, and another three antiepileptic drugs. The mean duration of epilepsy from onset to time of scanning was 12.2 ± 13.3 months (range 0.1–56 months).

### Ethics Statement

This study was approved by the Beijing Children’s Hospital Subcommittee on Human Studies. All study subjects and parents (or guardians) gave written informed consent prior to participation.

### Neuropsychological tests

Twenty-four children with epilepsy and 18 controls were administered the Wechsler Intelligence Scale for Children China-Revised (WISC-CR) test, which included FSIQ, verbal IQ (VIQ), and performance IQ (PIQ).

### fMRI data acquisition

We performed functional and structural neuroimaging in children with BECTS and healthy controls using a Siemens Trio 3T scanner at Beijing 306 Hospital. We acquired resting-state functional images using a single-shot, gradient-recalled echo planar imaging sequence (2000 ms repetition time, 30 ms echo time, 90° flip angle, 210×210 mm^2^ field of view, 64×64 in-plane matrix, 4 mm slice thickness, 0.8 mm interslice gap, 3.3 × 3.3 × 4 mm^3^ voxel size, and 30 transverse slices aligned along the anterior–posterior commissure). We instructed subjects simply to rest, not to think of anything in particular, and not to fall asleep. Subsequently, we acquired high-resolution T1-weighted anatomical images in sagittal orientation using a magnetization-prepared rapid gradient-echo sequence (2300 ms repetition time, 2.98 ms echo time, 9° flip angle, 240 × 256 mm^2^ field of view, 256 × 256 in-plane matrix, 1 mm slice thickness, 0.5 mm interslice gap, 1 × 1 × 1 mm^3^ voxel size, and 176 slices).

### fMRI data processing

Functional image preprocessing was carried out using the Data Processing Assistant for Resting-State fMRI (DPARSF; http://www.restfmri.net) [30], which synthesizes procedures in the Resting State fMRI Data Analysis Toolkit (REST; http://www.restfmri.net) [31] and Statistical Parametric Mapping (SPM8; www.fil.ion.ucl.ac.uk/spm). The first 10 images were excluded to ensure steady-state longitudinal magnetization, and the remaining images were then corrected for temporal differences and head motion. After subject selection, neither translation nor rotation parameters in any given data set exceeded ±3 mm or ±3°. We warped the functional images into a standard stereotaxic space at a resolution of 3 × 3 × 3 mm^3^, using the Montreal Neurological Institute (MNI) echo-planar imaging template, and then we spatially smoothed them with a 6-mm full-width half-maximum isotropic Gaussian kernel. Finally, we removed linear trends from the time courses, performed temporal band-pass filtering (0.01–0.08 Hz), and regressed out 9 nuisance signals (global mean, white matter, cerebrospinal fluid signals, and 6 head-motion parameters).

### ALFF analysis

ALFF was defined as the averaged square root of activity in the low-frequency band (0.01–0.08 Hz) [18]. ALFF value of each voxel was standardized by dividing the full-brain mean ALFF values. Two-sample t tests were used to compare the differences in ALFF between the patients and controls. Using the REST AlphaSim program, a corrected significance level of P < 0.01 was obtained by clusters with a minimum volume of 1808 mm^3^ and individual voxel height threshold of P < 0.05.

### GCA

A cluster showing abnormal ALFF was identified in rolandic area, the peak voxel (with a 3-mm radius) in this cluster was used as the seed region for the following GCA. The voxel-wise coefficient GCA [32] was performed in the whole brain using REST-GCA, a plug-in implemented in REST software [33]. We applied bivariate coefficient GCA to investigate the causal relation between the EZ and each voxel in the entire brain [21]. To explore the driving effect from the seed (EZ) to whole brain, one-sample t tests were performed for the causal effects within each group, with an uncorrected significance level of P < 0.05. The resulting maps of the two groups were combined and taken as a causal effect mask. Two-sample t tests were performed on the causal effects between groups within the causal effect mask with an AlphaSim-corrected significance level of P < 0.05 (height threshold, P < 0.01; extent threshold k = 432 mm^3^). The analysis for the driving effect from whole brain to seed (EZ) was performed in the same way as the seed (EZ) to whole brain.

To explore whether the neuroimaging measures correlate with the disease features in BECTS children, Pearson correlation was performed between causal effect/ALFF and disease duration/IQ scores at the peak voxel of clusters from the between-group analysis.

## Results

### Neuropsychological testing

Table 1 shows the demographic, clinical and neuropsychological characteristics of the patients with BECTS and healthy controls. FSIQ of the patients with BECTS was significantly lower than that of the control group (t = -2.39, P = 0.024). The PIQ scores did not differ significantly between the patients with BECTS and the healthy controls (t = -1.55, P = 0.13). The VIQ scores in the children with BECTS were significantly lower than those in the control group (t = -2.49, P = 0.021).

**Table 1:** Demographic, clinical, and neuropsychological characteristics of BECTS patients and healthy controls.

Characteristic	Patients	Controls	t/χ^2^	P value
Age (years)	9.8±1.5	10.0±1.5	-0.645	0.522^a^
Sex (female/male)	19/18	11/14	0.323	0.570^b^
FSIQ	90.50±9.22	100.56±15.94	-2.39	0.024^a^
VIQ	91.75±8.24	103.44±18.64	-2.49	0.021^a^
PIQ	91.17±11.37	96.94±12.77	-1.55	0.130^a^
Duration (months)	12.2±13.3	NA		–

The intelligence quotient (IQ) scores in patients and controls were based on the results of 24 and 18 participants, respectively. FSIQ = full scale IQ; VIQ = verbal IQ; PIQ = performance IQ. NA, not available.

^a^Two-sample *t* test.

^b^χ^2^ test.

### Between-group analysis of ALFF

**Fig 1:**
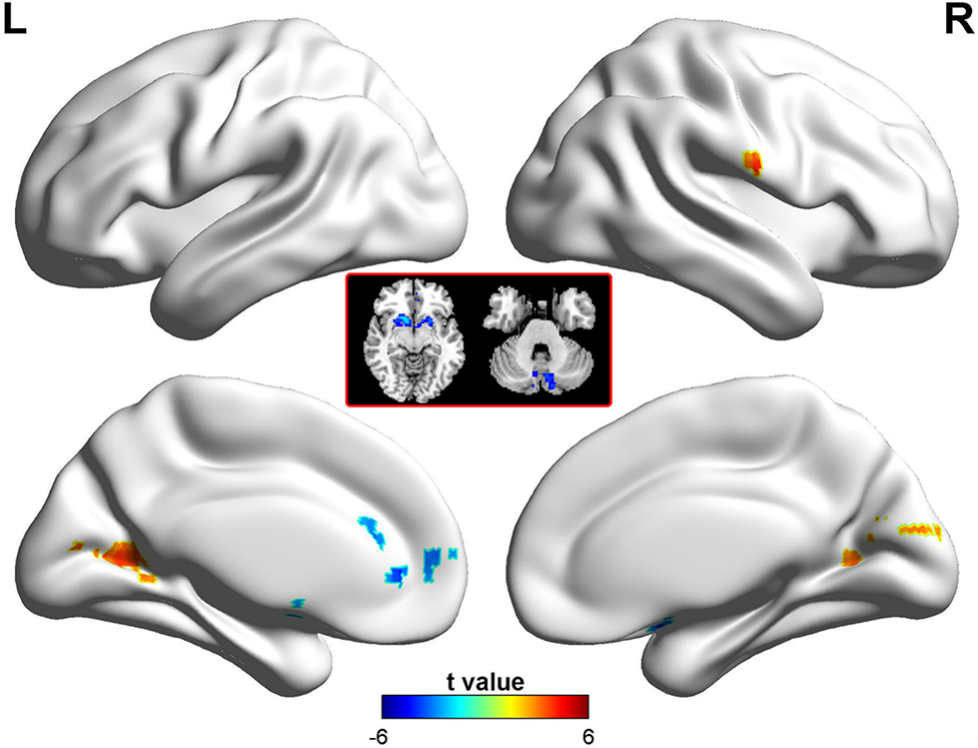
Brain regions showing abnormal ALFF in patients with BECTS. The warm (red) and cold (blue) colors represent higher and lower ALFF, respectively, in patients compared with controls (P<0.05, corrected). Color bar represents t values.

As compared with the healthy controls, the patients with BECTS showed significantly increased ALFF in the right postcentral gyrus and bilateral calcarine (P < 0.05 corrected) (Table 2 and Fig 1). Brain regions showing decreased ALFF included the left anterior cingulate cortex (ACC), bilateral putamen/caudate and left cerebellum (P < 0.05 corrected) (Table 2 and Fig 1).

**Table 2:** Regions showing abnormal amplitude of low-frequency fluctuation in patients.

Brain region	MNI	BA	Cluster size (mm^3^)	Peak t value
Postcentral gyrus R.	63, -9, 18	3	1323	4.23
Calcarine L.	-18, -72, 9	17	3024	3.94
Calcarine R.	21, -72, 15	18	2268	4.07
ACC L.	-6, 42, 3	32	2592	-4.63
Putaman/caudate L.	-21, 15, -9	NA	1728	-3.87
Putaman/caudate R.	18, 18, -9	NA	3186	-5.30
Cerebellum L.	-6, -78, -48	NA	7992	-4.56

ACC = anterior cingulate cortex; L = left side; MNI = Montreal Neurological Institute; R = right side;NA, not available.

### Voxel-wise GCA

#### Seed-to-whole-brain analysis

**Fig 2:**
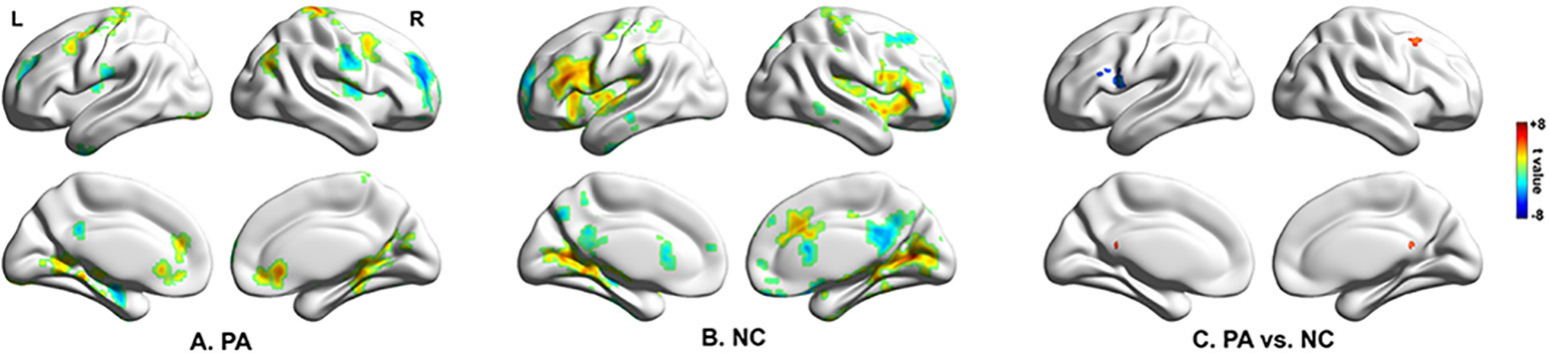
Voxel-wise GCA. (A) Regions showing significant causal effect with the seed in patients. (B) Regions showing significant causal effect with the seed in controls. (C) Regions showing abnormal causal effect with the seed in patients compared with controls. Color bar represents t values. NC = normal control; PA = patient.

Widespread cortical and subcortical structures were driven by the seed region in patients with BECTS (Fig 2A). The pattern in the healthy controls (Fig 2B) was clearly distinct from that in the patients. Compared with healthy controls, the patients showed an increased driving effect from EZ to the right medial frontal cortex (BA8) and posterior cingulate cortex (PCC) (Fig 2C and Table 3), and a decreased causal effects from EZ to the left inferior frontal gyrus (BA9/44/45/46) (Fig 2C and Table 3).

**Table 3:** Regions showing abnormal causal effect with epileptogenic zone in patients (seed-to-whole-brain).

Brain region	MNI	BA	Volume (mm^3^)	Peak t value	BECTS	NC
MFG R	45, 12, 51	8	1620	4.02	3.53*	-2.27*
PCC	0, -48, 18	23	459	3.75	1.22	-3.83*
IFG L	-57, 6, 18	44/9	2133	-4.45	-3.79*	2.62*
IFG L	-39, 21, 21	45/46	1053	-3.67	-0.15	4.43*
WM	-42, -3, 18	NA	1188	-3.98	-3.49*	2.32

The last two columns show the t value of the corresponding peak voxel within the patient and control group, respectively. Values with an asterisk show that the mean causal effect of the corresponding cluster is significantly different from zero. BA = Brodmann’s area; IFG = inferior frontal gyrus; L = left side; PCC = posterior cingulate cortex, R = right side; MNI = Montreal Neurological Institute coordinate; NC = normal control; WM = white matter;NA, not available.

#### Whole-brain-to-seed analysis

Whole-brain-to-seed analysis showed that there was no abnormal positive or negative driving effect from whole brain to seed in the patients with BECTS.

#### Correlation analysis

**Fig 3:**
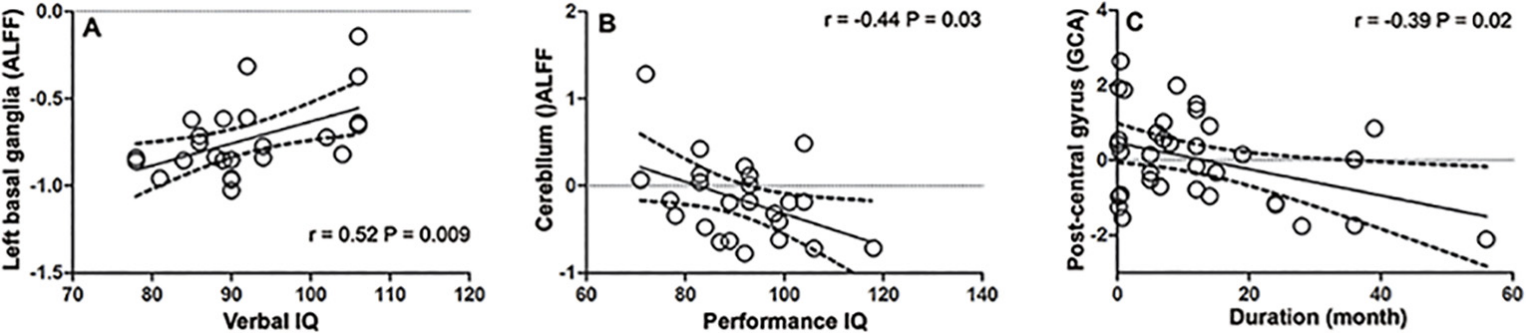
Correlation between fMRI measures and cognitive estimation or disease duration. (A) A positive correlation was identified for ALFF values in the putamen/caudate and VIQ scores. There were four children had same scores of verbal IQ(90), 4 points overlapping together, so there were only 22 points in the this figure. (B) The cerebellum and PIQ scores showed a negative correlation. (C) The causal effect from EZ to the left inferior frontal gyrus was negatively correlated with disease duration.

A positive correlation was identified for ALFF values in the putamen/caudate and VIQ scores (r = 0.521, P = 0.009) (Fig 3A). In contrast, the cerebellum and PIQ scores showed a negative correlation (r = −0.441, P = 0.031) (Fig 3B). The causal effect from EZ to the left inferior frontal gyrus (r = −0.393, P = 0.02) was negatively correlated with disease duration (Fig 3C). These associations were absent in the healthy controls.

## Discussion

In the current study, we localized the possible EZ of BECTS, and characterized the causal effect between EZ and the whole brain. We found that the possible EZ of BECTS not only showed abnormal increased local activity but also had abnormal driving effects to other brain areas.Findings of correlation analysis suggested that these disrupted brain activities were related to the abnormal cognitive functions.

This study identified that ALFF pattern in children with BECTS was altered compared to that in healthy controls.We found increased ALFF in the right postcentral gyrus, a presumed location of the generator of the epileptic discharges as supported by the findings of previous electroencephalography–fMRI studies [34–37]. We propose that the occipital activations (bilateral calcarine cortex) detected in our study might reflect some internally evoked secondary response of the brain to the initial spike event.

We consider decreased ALFF in the ACC, putamen/caudate and cerebellum to be responsible for functional impairments in cognitive processes in children with BECTS.

The ACC is involved in attention and concentration [38]. fMRI demonstrates ACC activation in executive control of attention [39], and conflict monitoring in the engagement of cognitive control [40]. One recent fMRI study of sustained attention demonstrated bilateral ACC activation, and a correlation between worse performance and lower BOLD signal in the ACC [41]. Moreover, a novel conflict monitoring task has been used to assess the effects on cognitive control of excitotoxic lesions in the ACC in rats. The animals with ACC lesions had difficulty in adjusting cognitive control [42]. Children with BECTS have sustained attention difficulties [7,43–45]. We infer that this decreased ALFF in the ACC may explain the attention deficit that is a common symptom in BECTS.

A particularly interesting finding in the current study was decreased ALFF in the putamen/caudate in children with BECTS, and a positive correlation was seen between ALFF values in the putamen/caudate VIQ score. The involvement of the basal ganglia has been demonstrated in focal and generalized epilepsy [46–48]. The basal ganglia were previously thought to be primarily involved in motor control, however, more recent evidence from neuropsychological and neuroimaging studies suggests that the basal ganglia also support language processing [49]. The striatal dopaminergic system plays an essential role in grammatical processes that form the core of human language [50]. The putamen plays a special role in reading but this is likely to vary with individual reading preferences and strategies [51]. Dysfunction particularly related to language has been reported in BECTS [3–6,52–54]. In the current study, we found that children with BECTS had a significantly lower VIQ score, which agrees with previous studies [55,56]. In the current study, there were decreased ALFF values in the putamen/caudate in children with BECTS, along with a correlation between these decreased ALFF values and VIQ scores. These results suggest that abnormal spontaneous neural activity in the basal ganglia may play a role in BECTS-related language dysfunction. One recent study found children with BECTS demonstrated significant putamen hypertrophy [57]. This finding, with combined our results, suggests that a structural abnormality underlies the functional abnormality in basal ganglia in BECTS. Additionally, the altered structural features in basal ganglia was also reported in children with absence seizures[58] As both absence epilepsy and BECTS are childhood onset and most patients of them become seizure free after adulthood, we speculate the basal ganglia may be a common target for epileptic patients with abnormal neurodevelopment.

Like basal ganglia, decreased ALFF values were also found in the cerebellum in children with BECTS. The involvement of the cerebellum in a wide range of cognitive functions has been found in many studies [59–61]. In addition, decreased ALFF values in the cerebellum were found to be negatively correlated with PIQ scores in children with BECTS. Whether these decreased cerebellar ALFF values were part of a compensatory mechanism limited progression of cognitive decline in BECTS remains to be determined in longitudinal studies.

After we identified the EZ (postcentral gyrus) by ALFF, we utilized coefficient-based GCA to obtain information about the flow directions and magnitude between the EZ (postcentral gyrus) and the whole brain. Unlike the residual-based GCA, this novel GCA method can characterize not only the positive causality but also negative causality. These two kinds of causalities may represent inhibitory and excitatory effect in physiology and have shed new light on the pathophysiology of several disorders [21,62,63]. Here, the predominant finding was that the abnormal causal effect in BECTS is unidirectional (seed to whole brain), without abnormal feedback (whole brain to seed). Compared with controls, patients demonstrated a decreased driving effect from the EZ to the inferior frontal gyrus (BA44/9, BA 45/46). Furthermore, we found that the abnormal driving effect from the EZ to the inferior frontal gyrus (BA 45/46) in patients was negatively correlated with disease duration. The traditional Broca’s area usually refers to the pars triangularis (BA 45) and pars opercularis (BA 44) of the inferior frontal gyrus. Broca’s area is one of the essential nodes in the language network. Evidence shows that BA 45 is implicated in semantic processing, while BA 44 is involved in phonological and syntactic processing [64]. Recent studies have demonstrated reduced functional connectivity between the sensorimotor network and the inferior frontal gyrus (Broca’s area) in children with BECTS [22,23], although the directions of information flow have been overlooked. For the first time, we identified a decreased driving effect from the EZ to Broca’s area in children with BECTS, and this agrees with the decreased VIQ. Our results provide new evidence that epileptiform activity in BECTS may cause language impairment.

In the current study, the ipsilateral medial frontal cortex (BA8) and PCC showed an increased causal effect driven by the right postcentral gyrus. The medial frontal cortex has previously been implicated in action selection/outcome monitoring, behavioral adjustments and learning [65–67]; all of which are important in complex cognitive tasks such as language. The PCC is the main hub within the default network whose activation is greater at rest than during tasks [68]. Several studies have found decreased resting-state connectivity within the default network in patients with epilepsy [69–72]. Oser and coworkers [73] have provided evidence of abnormal functional integration of the default network in BECTS. We speculate that the increased causal effects driven by the EZ to the medial frontal cortex and PCC are compensatory reallocations for the neural system to compensate for cognitive deficits in BECTS. Future studies are needed to confirm this speculation.

The current study had several limitations. First, it was not a longitudinal survey, therefore, it is unknown if the ALFF values and causal effect alterations will normalize with seizure remission in BECTS. Second, although only 4 children received antiepileptic drugs, the effects of medications cannot be fully excluded. Ideally, these results need validation in drug-naïve patients. Third, we did not simultaneously perform electroencephalography during fMRI, thus we did not know to what extent the functional alteration was caused by interictal discharges. Fourth, clinically, the identification of a real EZ should be based on a comprehensive evaluation, including video-EEG, clinical characteristics or even surgery. Although previous studies found ALFF significantly correlated with epileptic activity, the ‘potential’ EZ found here through ALFF still need further validation. Fifth, the maintaining of normal brain function may be largely dependent on the interaction between multiple networks. To estimate the effect of epileptic network on brain function, a network based analytic strategy may be adopted as previous epilepsy studies [74].

In conclusion, we characterized the abnormal local activity and causal connectivity in patients with BECTS using resting-state fMRI data. The findings from ALFF suggested that the inferior part of postcentral gyrus may be the EZ of BECTS, and the putamen/caudate and cerebellum play an important role in the cognitive dysfunction of BECTS. GCA indicated a decreased driving effect from the EZ to Broca’s area in children with BECTS, which was negatively correlated with disease duration, giving new evidence that epileptiform activity in BECTS may cause language impairment. Our findings provide neuroimaging evidence of the neuropathophysiological mechanisms underlying the cognitive impairments in BECTS.
